# Sibling Competition and Conspicuousness of Nestling Gapes in Altricial Birds: A Comparative Study

**DOI:** 10.1371/journal.pone.0010509

**Published:** 2010-05-06

**Authors:** Juan J. Soler, Jesús M. Avilés

**Affiliations:** Departamento de Ecología Funcional y Evolutiva, Estación Experimental de Zonas Áridas, Almería, Spain; Roehampton University, United Kingdom

## Abstract

**Background:**

Nestlings of altricial birds capture parents' attention through conspicuous visual displays, including exposure of their gape coloration which informs parents about their level of need, competitive ability or health; information that parents use for deciding food allocation among their offspring. Thus, because nestlings compete with nest mates for parental care, nestling conspicuousness is expected to increase with level of sibling competition along bird phylogeny.

**Methodology/Principal Findings:**

We test this prediction by jointly using information of brood reduction, clutch size and duration of nestling period as proxies for intensity of sibling competition, and visual models that assess detectability of nestlings by adult birds. As predicted, we found a positive association between nestling conspicuousness and intensity of brood reduction, while clutch size and duration of nestling period did not enter in the best models. Level of brood reduction was positively related with the achromatic component of nestling conspicuousness and body mass was negatively related with the chromatic component.

**Conclusions:**

These associations are in agreement with the hypothesis that sibling competition for parental attention has driven the evolution of visual nestling conspicuousness in a context of parent-offspring communication in altricial birds.

## Introduction

The extraordinary variation in the patterns of nestling coloration in birds has largely attracted the attention of evolutionary biologists. Functional hypotheses suggest advantages of nestling designs with colors that for instance enhance crypsis, prevent water loss, and allow a proper thermoregulation and an easy recognition by parents (reviewed in [1]). Perhaps the most fascinating evolutionary questions related to nestling coloration are associated with its prominent role in parent-offspring communication. Coloration may inform to parents on nestling level of need [2], and/or phenotypic quality [3]–[8]; and parents would use that information for adjusting parental effort [2], [6], [9]–[14].

Theoretical models suggest that the form and intensity of begging displays have evolved not only to communicate nutritional requirements and/or health status to parents, but also as a mechanism for competing against siblings to gain a greater amount of parental attention [15], [16]. Nestlings would thus compete with nest mates for attracting parental care by showing conspicuous begging displays to their parents. Therefore, in an inter-specific comparison, species exposed to higher levels of sibling competition are expected to evolve more conspicuous begging displays. In a comparative study, Briskie et al. [17] showed that loudness of nestling begging calls (i.e., one conspicuous component of nestling begging display) increases as the relatedness amongst the members of a brood declines. Because competition for parental attention is expected to be higher among unrelated nest mates [18], [19], Briskie et al. [17]'s results provided support for the sibling competition hypothesis.

Evidence supporting the sibling competition hypothesis for begging traits is however scarce and weak for nestling coloration despite its prime role in parent-offspring communication (see above). Gaping structures (i.e., flanges and mouth cavity) are the most conspicuous visual traits that unfeathered nestlings display to their parents, although skin color at other body parts may also reveal aspects of nestling quality [6], [7], [11]. Conspicuousness of colored begging traits would facilitate parental discrimination of nestlings [20]. It, thus, follows that conspicuousness should positively covary with level of sibling competition. In a first test of the sibling competition hypothesis Kilner and Davies [21] did not find evidence of covariation between clutch size and the size and coloration of begging structures in a set of 31 passerines. Clutch size was used as a species-specific proxy of sibling competition in the nest based on the assumption that for a similar amount of parental feeding effort sibling competition would be higher in nests with larger broods. In a subsequent test, Kilner [22] found partial support for the sibling competition hypothesis since redness of the mouth was positively related to the degree of extra-pair paternity (used as a proxy of the degree of sibling competition) among open-nester species, but not among hole-nester species. In these two studies, however, differences in nestling coloration were estimated by human eyes based on video images and/or descriptions of the nestling mouth colors which were drawn from the literature [21], [22]. It is now well established that birds have UV photoreceptors that may alter their perception of colors that humans see as red, orange or yellow [23], [24]. Also, begging structures of some altricial birds markedly reflect in the UV part of the spectrum which is blind to humans [25], [26]. Furthermore, it has been shown that nest luminosity affects the efficiency of mouth designs of nestlings attracting parental feeding attention [10], [27]. Indeed, flanges of hole nestling birds are globally more conspicuous at their natural luminal environments than those of open nesting species when perceived by a bird eye [26]. Finally, it has been recently shown that nestling gape color designs differ in relation to parental visual system [20]. Therefore, any attempt of assessing conspicuousness of begging color traits should account for nestling coloration, the contrast and luminal environments in which this visual display is perceived (i.e., the nest), and the perceptual capacities of the intended receiver of the display (i.e., the parents) [20], [26].

In this paper, we revisited the sibling competition hypothesis for the visual conspicuousness of gaping structures in a comparative approach. We estimated visual conspicuousness of gaping traits with a visual model approach that assessed conspicuousness of nestlings as would be perceived by birds. In a previous study we have controlled for a possible role of sibling competition on the relationship between nestling conspicuousness and nest type by using clutch size as a proxy for the level of sibling competition [26]. We found that whole nestling (i.e. taking together colorations of mouth, flanges, breast and head) achromatic contrasts with nest background increased with clutch size, while chromatic and achromatic contrasts between different body parts were unrelated to clutch size [26]. In that previous article, however, we did not explore the relationships between clutch size and contrasts of nestling gapes with the nest, which are the most visually conspicuous nestling traits used during begging displays in altricial birds. Moreover, the merely use of clutch size as a surrogate for sibling competition is debatable. Although at every nest visit there are more rivals for the carried prey items, nestlings of species with large broods do not always experiences lower survival than those of species with small broods. Indeed, the main cause of nestling mortality in non-depredated nests is starvation (or related diseases) (e.g., [28]), which is considered the results of sibling competition for attracting parental feedings [29]. Here, in addition to clutch size, we used level of brood reduction in successful nests (those that escaped of predation and produced at least one fledgling) as a second proxy of level of sibling competition for parental attention. Finally, because duration of nestling period predicts the level of sibling aggression [30], we also included in the analyses the duration of nestling period as a third variable, possibly reflecting level of sibling competition, to explain interspecific variation in gape conspicuousness of nestlings birds. Hence, here we have explored the association between conspicuousness of nestling gapes (i.e., mouth and flanges) and three different variables related to level of nestling competition for parental attention.

## Materials and Methods

Nestling coloration, visual backgrounds at the nests and light environments were measured in the surroundings of Guadix (37°18′N, 3°11′W), south-east Spain, in March–June 2005–2007. The predominant habitat includes cultivated areas with some remains of holm oak forest, grows of almond trees, and olive trees and other tree crops in irrigated areas surrounding villages. We collected data on nestling coloration on 483 nestlings of 21 species included in 13 families (File S1). Hole-nesting species were mostly located within nest-boxes recently (2003–2005) installed. All sampled chicks were measured at a standard relative age during their ontogeny (i.e., when they were in the first third of its normal nestling development; with closed eye and no pin feathers). We have not observed any brood reduction in the sampled nests before taken color measurements, and avoided sampling nestlings in apparent poor physical conditions (i.e., close to die or runts).

Research has been conducted according to relevant national (REAL DECRETO 1201/2005, de 10 de octubre), guidelines. Nestlings were handled under the authorization of The Junta de Andalucía – Consejería de Medio Ambiente (permits No. SCFFS-AFR-CMM, SGYB-AFR/CMM). To avoid nest abandonment, we always left at least one chick in the nest while collecting reflectance spectra. Nestlings were returned to their nest before fifteen minutes from removal, and subsequent visits to these nests confirmed us that our manipulation had no effect on nestlings.

### Spectral reflectance of nestlings

Reflectance spectra (300–700 nm) of nestlings were recorded using an Ocean Optics equipment [S2000 spectrometer connected to a deuterium-halogen light (D2-W, mini) by a coaxial reflectance probe (QR-400-7-UV-vis) and the OOIBase32™ operating software (Ocean Optics, Inc. Dunedin, FL, USA)]. Reflectance was always measured with the probe placed at a constant distance and reaching the nestling at 45°. Measurements were relative and referred to a standard white (WS-2) and to the dark, which were calibrated before measurement of each nestling. To standardize ambient light during data collection all measurements were taken within a portable hide with opaque wall set in the surrounding of the nests. Mouth color was measured by gently keeping the gape open and introducing the probe to the centre of the upper mouthpart. Flanges were measured maintaining nestlings with the mouth almost closed, and placing the probe on the angle of the mouth-flanges, thus, avoiding confusion with mouth coloration. All color measurements were repeated three times per nestling trait and, since we have previously demonstrated that measurements of nestling coloration were repeatable [20], mean values per nestling were calculated and used in the analyses. Average reflectance spectra of gaping traits of the considered species except that of house sparrow (*Passer domesticus*) are displayed in Fig. A1 of Aviles *et al.* [26].

### Spectral reflectance of nest background and irradiance spectra

Spectral reflectance of nest backgrounds was estimated from nest material collected in active nests of (i) species that build no nest at all and whose nestlings only can contrast with the substrate (e.g. owls, falcons and coraciiforms); (ii) species that build a nest cup mainly with dry grass (e.g. Turdidae or magpies (*Pica pica*)); (iii) species that line the nest mainly with thin shrub or tree branches and do no provide additional material to line the nest (e.g. pigeons); and (iv) species that line the nest with wool or feathers (e.g. Corvids (except magpies) shrikes, swallows or tits).

When nest size made it possible, the entire nest was collected and saved in a plastic bag. For species having big nests, however, only a representative fraction of the nest lining was collected and preserved in plastic bags. Entire nests or parts of the nest line were always collected from active nests once nestlings had fledged. When arriving to the laboratory we measured nest line coloration with an Ocean Optics spectroradiometer using the above equipment and specifications for nestlings. All measurements were taken in dark. For every collected nest, the material of the nest line was disaggregated and representative materials laid flat trying on a matte black cardboard for measurements. We obtained representative reflectance spectra of nest background in the four different types of nests by sampling a total of 29 nests of 18 species. Ten readings were taken at every nest. This technique provided repeatable measures of nest color for the three first PC scores of a PCA summarizing 98.62% of whole variation in nest color (PC1: *R* = 0.54, *F*_28,268_ = 12.78, *P*<0.0001; PC2: *R* = 0.44, *F*_28,268_ = 29,61, *P*<0.0001; and PC3: *R* = 0.64, *F*_28,268_ = 18.57, *P*<0.0001). Therefore, mean values per nest type were calculated based on mean values of species within the same group. Average reflectance spectra of nest background in the four types of nests in which model calculations were based are displayed in Fig. A2 in Aviles *et al.* [26]. Species classification regarding nest type is shown in File S1. However, due to genetic relatedness and shared environment, nestlings from the same nests would be more similar to each other than to non-related nestlings reared in different nest environments. Consequently, assumed errors for species estimations with a few sampled nests could be relatively higher than that expected from the number of nestlings sampled.

Ambient light measurements in the nests were collected during the morning (09.00–11.00 am), when parental provisioning to the nests is maximal. Briefly, we used a cosine-corrected fiber-optic probe (P400-1-UV-VIS, Ocean Optics) with a 180° angle of acceptance and a measurement surface of 6 mm in diameter (CC-3-UV, Ocean Optics). The spectrometer was calibrated with light source of known color temperature (LS-1-CAL; Ocean Optics). We measured the ambient light at open areas (ten readings) and inside the nest-boxes, close to the entrance (ten readings), with the measurement surface oriented to the skyward or roof respectively and the probe held perpendicular to the ground. We transformed irradiance readings into photon units as described by Endler [31] and calculated mean values across open and hole nests to obtain average irradiance spectrum in these two nest environments. This is justified by the high repeatability of the PC1 scores of a PCA summarizing 96.34% of the variation in nest color irradiance at these two nest environments (*R* = 0.98, *F*_1,23_ = 272.30, *P*<0.0001). Average irradiance spectra in open and hole nests in which model calculations were based are displayed in Fig. A3 in Avilés *et al.* [26].

### Parental visual system

Information on vision type only exits for few species (7 out of 21 species) but this span most families (11 out of 14 families) included in our sample (File S1). The VS type is the ancestral state in birds although the UVS state has evolved independently at least four times [32]. However, evidence coincides that most of Passeridae are of the UVS type ([33]–[35] with the exception of members of the groups Corvidae and Tyrannidae [32]. Furthermore, no splits in the type of vision have so far been reported within a bird family [32], [36] which suggests that vision type has a strong phylogenetic inertia in birds [23]. Therefore, we used cone sensitivities of a typical UVS bird for all Passeridae with the exception of the members of the superfamily Corvoidea (Corvidae and Laniidae) that were modeled as a VS species. The remaining sampled species were treated as VS birds (File S1). Trying to detect how important is the assumption of considering different species as UV or UVS in our results, calculations were repeated for every sampled species and nestling trait by using both spectral sensitivity data and cone proportions of a typical UVS and VS bird.

### Avian color space modeling

We used the visual model developed by Vorobyev and Osorio [37] as developed for the tetrachromatic visual system of birds in its log form [38]. The model has been demonstrated to describe visual discrimination in birds [37], [39], and, recently, it has been successfully incorporated in comparative studies of bird coloration [26], [40]–[42]. The model establishes chromatic distance Δ*S* which describes the color contrasts between two colored patches as:
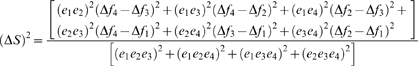
(1)where Δ*f*_i_, is the log ratio of the quantum catches for cone *i*, for chick trait A and B and e_i_ is the signaling noise for each cone class i.
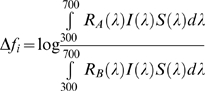
(2)where *R_A_*(*λ*) represents the reflectance of the patch A, *R_B_*(*λ*) is the reflectance of the patch B, *I*(*λ*) is the spectral irradiance of the illuminant, and *S*(*λ*) is the spectral sensitivity of the receptor *i*. The model application used here involves the calculation of color distances Δ*S* within the visual ‘space’ of the parent birds. Essentially, different colors that appear similar to a signal receiver (either because of the nature of their visual system or an absolutely small difference in the reflectance spectra of the colors) result in small Δ*S* values, while those that have high chromatic contrast have large Δ*S* values. Following recently published literature, we used spectral sensitivity data from the blue tit *Cyanistes caeruleus* and the peafowl *Pavo cristatus* as representative of the UVS and the VS system respectively (e.g., [40], [42]). Further, since ratios between different cone types for the 22 species reported in Hart [43] do not significantly differ between UVS and VS species [20] for the noise calculations we used constant cone proportions of 1, 1.92, 2.68, and 2.7 for UVS [35] and, 1, 1.9, 2.2, and 2.1 for VS [44], and assumed that the signaling noise for each cone was independent of light intensity
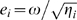
(3)where *ω* is the Weber fraction (taken as 0.05) and *η_i_* is the relative density of the cone class i on the retina.

It is well known that birds can use achromatic (brightness) contrasts in discriminatory tasks (reviewed in [45]). In birds double cones are assumed to be responsible of achromatic visual detection (e.g., [46], [47]). Therefore, we calculated receptor signals (i.e. achromatic Δ*Q* contrasts between two colored patches) for double cones using the formula above and the spectral sensitivities for double cones in blue tit [35] and peafowl [44].

### Sibling competition

As reported above the level of sibling competition for parental attention is expected to covary with at least three different life-history traits, namely clutch size, duration of nestling period and level of brood reduction. As in previous articles (e.g., [21], [26], [30]), we used clutch size (as reported by Cramp [48]) as a proxy for brood size in the expectation that sibling competition was stronger for larger brood sizes. We relied on clutch size because brood sizes and clutch sizes are tightly correlated across bird species [30], and information of brood size was not available for some species in our data set.

Duration of nestling period is known to be positively related to intensity of sibling aggression in bird species in which competition for parental investment is violent [30]. Thus, duration of nestling period could also be positively related to intensity of sibling competition for parental attention because long nesting period give more chance to food scarcity [30]. Here we deal with species in which competition for parental attention is not violent and retrieved information on duration of nestling period from Perrins [49].

Species-specific level of brood reduction, estimated as the average percentage of hatchings that successfully reached the fledging stage in successful nests, is a direct measures of probability of dead that each sibling in a brood experienced during the nesting phase, and directly reflects intensity of sibling rivalry for parental attention [29]. However, brood reduction might be the result of parental manipulations such as hatching asynchrony and maternal effects, which allow a brood size adjustment to environmental conditions and/or create a competitive hierarchy that in some species, rather than increase, might decrease level of nestling competition [29], [50]. Even in that case, intensity of sibling competition for parental attention should be higher in species experiencing higher level of brood reduction, at least before brood adjustment takes place.

Because we were interested on natural level of brood reduction (i.e., excluding depredated nests), we used mean clutch size as a proxy of number of hatchlings, and estimated number of hatchlings that reached fledging stage from average fledging success of the species after excluding depredated nests. For most species we found information of level of brood reduction for more than one population and/or study year (see File S1), which allowed us to estimate repeatability of our estimates for a species. We found that among species variance in estimates of brood reductions was highly significantly larger than the within species variance (one-way ANOVA, F = 6.68, df = 20, 25, P<0.00001), which resulted in a moderately high repeatability (R = 65.4%). Thus, we used mean values of such estimations in our analyses. Literature used to estimate level of brood reduction and its average value per species are given in File S1.

### Comparative analyses

Taxonomic groups such as species cannot be considered statistically independent observations due to the confounding effects of common ancestry [51]. To control for the phylogenetic relationship among the sampled species we used phylogenetic generalized least square regression (PGLS) models [52], [53] as implemented in *R* statistical environment with the appropriated libraries (“ape”, “MASS” and “mvtnorm”) and additional unpublished function by R. Freckleton (University of Sheffield) (pglm3.3.r, available on request). We considered indexes of sibling competition (i.e. level of brood reduction, clutch size, duration of nestling period) as the independent variables in our analyses because the hypothesis tested is that sibling competition influences the evolution of nestling conspicuousness. Patterns of nestling conspicuousness differ between hole and non-hole nesting species in altricial birds [26]. In addition, body mass is related to duration of nestlings period and clutch size [54]. Therefore, we included information of nesting habits (i.e., hole vs non-hole) and body mass as reported by Cramp [48] as additional factors in our analyses. Distribution of chromatic and achromatic contrasts with the nest background and among the different body regions, as well as level of brood reduction and log-transformed clutch size, log-transformed body mass and log-transformed duration of nestling period did not differ significantly from normality (Kolmogorov-Smirnov tests, P>0.05).

The PGLS approach characterizes evolutionary changes along each branch of a phylogeny through the variance components of traits and controls for the non-independence among species by incorporating a matrix of the covariances among species based on their phylogenetic relationships [52], [53], [55]. The method applies likelihood ratio statistics to test hypotheses of correlated trait evolution and also to estimate the importance of phylogenetic corrections in the models [56]. We conducted all analyses setting the degree of phylogenetic dependence (λ) to the most appropriate degree evaluated for each model. Because we have not *a priori* expectation on the specific surrogate of sibling competition that could better explain interspecific variation in nestling conspicuousness we used Akaike's information criterion (AIC) [57] for model selection. Models with estimated corrected AICc's values not differing in more than two units with that estimated for the best model were considered equally explicative of dependent variables [57]. Final models were later tested in PGLS analyses to estimate the relative contribution of each factor in the models.

Our phylogenetic hypothesis was based on Livezey and Zusi [58] for the basal nodes and in Jonsson & Fjeldsa [59] for the upper nodes (File S2). We arbitrarily assigned all inter-node branch segments equal to one.

## Results

Specific levels of brood reduction, clutch size and duration of nestling period did not result significantly related to each other and body mass was negatively and positively related to clutch size (PGLS; beta(SE) = −0.28(0.09)) and to the duration of nestling period (PGLS, beta(SE) = 1.78(0.60)) (Table 1), respectively. Furthermore, none of these variables were related to nesting habits (Table 1). These results, on the one hand exclude problems of collinearity by the simultaneous use of these factors as independent variables in the same model, and validate the inclusion of body mass in the models as a variable necessary to control the effects of clutch size and duration of nestling period. On the other hand, the low covariation between variables hypothetically related to intensity of sibling competition (see above) would suggest that these factors might explain different portions of whole variance in sibling competition.

**Table 1:** Correlation matrix between variables used as proxies of intensity of sibling competition of the 21 species analysed.

N_(species)_ = 21	Clutch size	Body mass	Duration of nestling period	Nesting habits
Brood reduction	R^2^_adj_ = −0.0002, P = 0.33	R^2^_adj_ = 0.0002, P = 0.33	R^2^_adj_ = −0.004, P = 0.33	F = 0.35, P = 0.55
Clutch size		R^2^_adj_ = 0.29, P = 0.007	R^2^_adj_ = 0.01, P = 0.27	F = 0.39, P = 0.51
Body mass			R^2^_adj_ = 0.28, P = 0.008	F = 1.29, P = 0.18
Nestling period				F = 2.53, P = 0.07

Values are phylogenetically corrected by means of PGLS analyses.

**Figure 1:**
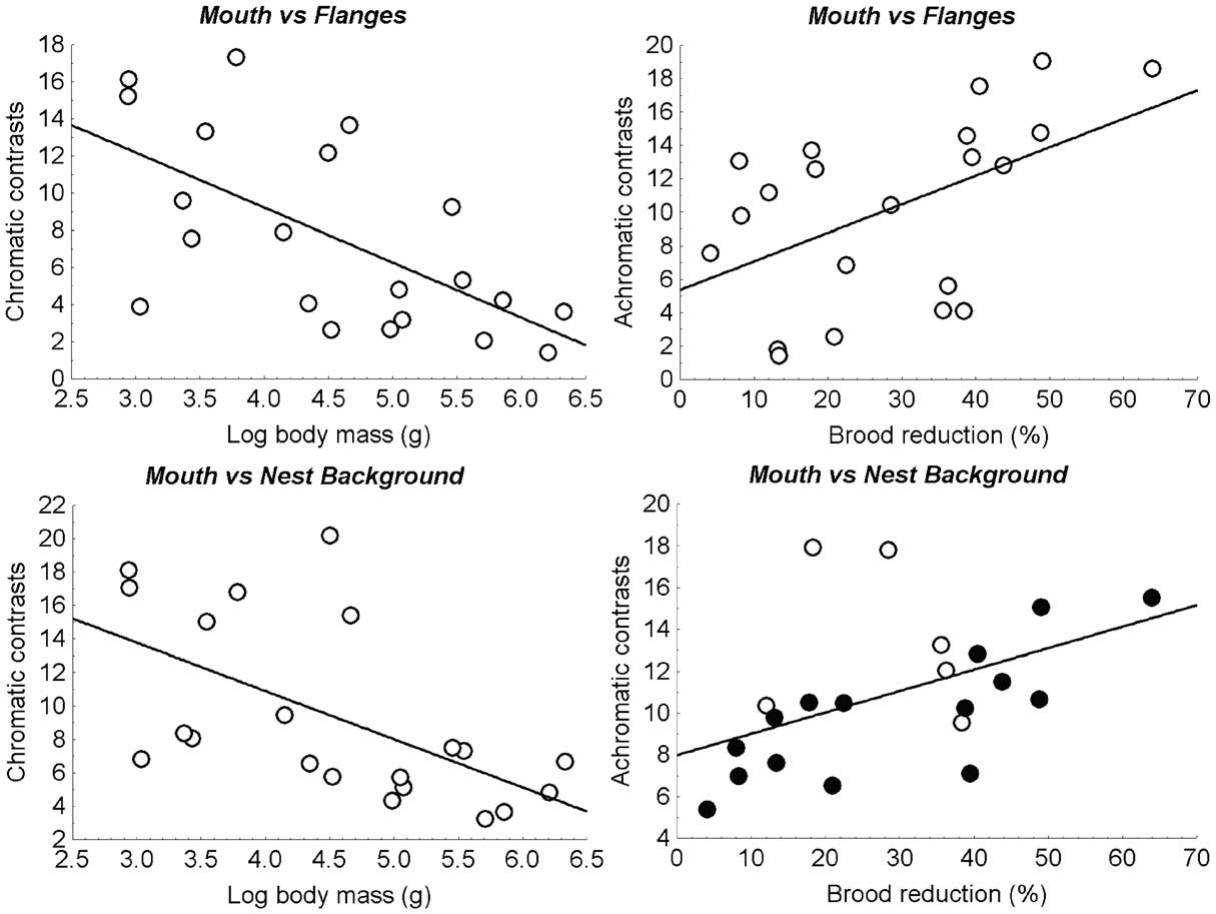
Relationships between chromatic and achromatric contrasts of nestling vocal traits against each other (i.e. mouth vs flanges) or against the nest background and level of brood reduction and body mass. Figure with filled (hole nesting) and empty (non-hole nesting) circles represent the effect of nesting habits explaining interspecific variation of achromatic contrasts of mouth vs background contrasts.

Level of brood reduction and body mass respectively explained achromatic and chromatic conspicuousness of mouth coloration when contrasting against nestling flanges (Table 2). Chromatic conspicuousness of flanges against the nest background was better explained by level of brood reduction, while achromatic contrasts were better explained by clutch size, nestling habits and body mass (Table 2). However, none of these factors explained a significant proportion of variance (Table 2). Species with larger values of achromatic contrasts between mouth and nest background were those nesting in holes and with higher level of brood reduction (Table 2). Finally, chromatic contrasts between mouth and nest background were explained by body mass (Table 2). All taken together, our results indicate that conspicuousness of nestling gapes structures were positively related to level of nestling competition as reflected by specific level of brood reduction and negatively related to body mass (Fig. 1).

**Table 2:** Best models explaining the relationships between chromatic and achromatic contrasts of nestling gape traits against each other or against the nest background and variables reflecting level of nestling competition (broodr: brood reduction; lgCS: log transformed clutch size; lgNP: log transformed nestling period) or those known to affect dependent and/or independent variables (hole: nesting habits (hole vs non-hole); lgBM: log transformed body mass).

Dep. variable	V(1)	V(2)	AICc	w_31_	Evidence ratio w_31_	PGLM (Model) R^2^_(adjusted)_(P)	Beta(SE) V(1), P	Beta(SE) V(2), P
Achromatic contrasts								
Mouth vs flanges	broodr		126.16	0.437	1.000	0.29 (0.007)	0.19(0.06), 0.007	
Flanges vs nest	hole		94.02	0.243	1.000	0.10 (0.09)	0.86 (0.99), 0.086	
	lgCS		94.85	0.161	1.511	0.05 (0.17)	−1.47 (1.04), 0.17	
	lgBM		95.93	0.094	2.590	0.01 (0.34)	0.46 (0.47), 0.34	
Mouth vs nest	broodr	hole	108.65	0.573	1.000	0.48 (0.001)	0.13 (0.04), 0.008	1.58 (1.07), 0.0039
Chromatic contrasts								
Mouth vs flanges	lgBM		110.61	0.380	1.000	0.29 (0.007)	−2.89 (0.96), 0.007	
	broodr	lgBM	111.96	0.193	1.968	0.33 (0.01)	−0.06 (0.04), 0.15	−2.87 (0.93), 0.006
Flanges vs nest	broodr		92.58	0.281	1.000	0.06 (0.14)	0.04 (0.03), 0.14	
Mouth vs nest	lgBM		115.85	0.376	1.000	0.18 (0.033)	−2.49 (1.09), 0.033	

Results are from Phylogenetic Generalized Least Squares analyses (PGLS). The best models were selected from the Akaike's criterion. We show the corrected AIC'\s value (AICc), the Akaike Weights estimated from the 31 possible models (W_31_), and the Evidence Ratios of each model. Furthermore, we also show percentage of variance (R^2^_(adjusted)_) explained by each model (i.e., those with AICc values differing less than 2 from the AICc value of the best model), as well as beta(SE) values associated to each of the independent variable in the model. When nesting habits (hole vs non hole) appeared in the tested models, effect size (+/− confidence intervals (95%)) estimated with F-values from the ANOVA code in PGLS were reported.

## Discussion

Our results confirm the predicted key role of sibling competition for attracting parental attention on the evolution of visual conspicuousness of nestlings in altricial birds. We have found a positive relationship between level of brood reduction and the achromatic components of contrasts between gape structures (i.e., mouth vs flanges) of nestlings, and between mouth and background colors (Table 2). These relationships were controlled for possible confounding factors due to common ancestry and for parental visual system and nesting habits. Below, we discuss these results under the hypothesis that sibling competition for parental attention at the nest drives the evolution of visual nestling conspicuousness.

In accordance with previous works [21], [26], [60], we predicted a positive association between clutch size and conspicuousness of nestling gapes. Clutch size, however, did not enter in the best models explaining significant proportion of variance of nestling conspicuousness. In a previous article exploring the effect of nest luminosity (i.e. nesting habits) on nestling coloration we did find a positive association between clutch size and nestling conspicuousness after controlling for the effect of nesting site [26]. Here, results from most PGLS models that included clutch size and nesting habits as independent variables explaining chromatic and achromatic conspicuousness of nestling gapes also detected the predicted positive relationship (PGLS, beta(SE) associated to clutch size; chromatic contrasts: mouth-flanges = 3.46(2.27), flanges-nest background = 0.46(1.37); mouth-nest background = 3.63(2.39); achromatic contrasts: mouth-flanges = 3.87(3.05), flanges-nest background = −1.18 (1.04); mouth-nest background = 0.69(1.69)), but in none case P-values associated to clutch size reached statistical significance (P>0.13). In our previous work we only partially controlled for phylogenetic similarity by including species identity as random effects in the models, while here we relied in PGLS models that allowed a more appropriate phylogenetic control. These differences in the analytical approaches might explain different results. Clutch size entered occasionally in our best models explaining nestlings' conspicuousness, and in no case it explains significant proportion of variance. These results suggest a limited importance of brood size explaining the evolution of nestling gape coloration. An alternative explanation is that the relationship between clutch size and body mass masked the predicted effect of clutch size. We found that nestlings of smaller species had more conspicuous gapes (see results). Smaller species also laid larger clutches (see results) and, consequently, it is possible that the significant association between body mass and conspicuousness of nestling gapes were partially due to the negative association between clutch size and body mass.

We have found a negative association between body mass and nestling conspicuousness although we had not *a priory* prediction for the association between body mass and nestling color. Indeed, we used body mass for controlling clutch size and duration of nestling period in the analyses. However, it is possible that some kinds of constraints that differ for species of different body mass was responsible for the detected association between body mass and conspicuousness of nestling gapes. Evidence suggests that red and yellow colors of nestling gapes are costly to produce [1]–[3], [5]. Thus, it is possible that mouth coloration was constrained by physiological tradeoffs [1], and that the resolution of such tradeoffs differed for species of different size as they diverge in physiological requirements. Although it is speculative, it seems worth to further explore this hypothetical scenario in the future.

Duration of nestling period did not enter in our best models explaining conspicuousness of nestling gapes. However, duration of nestling period and body mass were negatively related, and this association might partially explain the detected association between nestlings conspicuousness and body mass (see Results). Duration of nestling period resulted positively related to intensity of sibling aggression in a interspecific comparative study [30]. This relationship was predicted since long nestling period should favor costly-aggressive competition because the substantial early investment involved in established dominance is more likely to be adequately compensated when brood mates cohabit and compete for parentally provided food for a long period [30]. Sibling aggressions, however, are not included in the begging display and are not directed to parents, but limit the effectiveness of begging by subordinated siblings [61]. Furthermore, conspicuousness of nestling gapes has likely evolved as a result of sibling competition for parental resources by appealing to some aspect of parental psychology, but not for sibling competition for establishing within nest hierarchy [1]. Therefore, the predicted positive relationship between the duration of nestling period and sibling competitive aggression may not apply when trying to explain conspicuousness of nestlings gapes as consequence of sibling competition for parental attention.

Brood reduction, estimated as percentage of nestlings that do not successfully fledge in non-depredated nests, entered in the best models explaining both chromatic and achromatic conspicuousness of nestling gapes. We thus used the narrow sense of brood reduction [62], which by definition refer to the “within-brood partial mortality that is due to sibling rivalry *per se*” (see [29], pg. 77). Brood reduction, in this sense, is assumed to be adaptive since fatal levels of sibling competition would trim brood size to an appropriated level if food turns out to be low [28], [62]. Thus, the detected positive association between level of brood reduction and conspicuousness of nestling gapes suggests that intensity of sibling rivalry for parental attention played a role in the evolution of nestling coloration. It should be note here that sibling rivalry does not only refers to competitive begging scrambles between sibs, but also to between-sibling rivalry mediated by the expression of traits that honestly signal the reproductive values of nestlings. Assuming parental preference to feed the most conspicuous gapes in their nests [3], [6], [63], and considering that among siblings variation in gape conspicuousness was, at least partially, under genetic control, processes of natural selection within the non-depredated nests would explain the detected interspecific association between intensity of brood reduction (i.e. natural selection) and gape conspicuousness.

The hypothesis tested refers to the conspicuousness of nestling traits when perceived by their parents, which may differ from estimates of nestling coloration from human vision, from image analyses, or from direct quantification of reflectance at different wavelengths. A more thorough understanding of the evolution of nestling coloration would thus require nestling conspicuousness assessment from the perspective of adult birds [26]. We assessed perception by adults of nestling color-traits by means of visual models that take into account most factors affecting visual perception [37], [38]. Perception by adults is appraised in these models by estimating visual contrasts, which determines detectability of a target object that is viewed contrasting with its natural background. As a background we have used nest material, but also flanges. Previous work has emphasized the role of flanges as mouth-contrasting traits that facilitated nestling detectability and, thus, entire design of gape structures (i.e., mouth and flanges) should enhance chick conspicuousness and, therefore affect parental decision of food distribution among siblings (see [1]). In accordance with this evolutionary scenario we have found that visual contrasts that quantified nestlings conspicuousness was predicted by variables related to intensity of sibling competition for parental attention. These results, therefore, are in agreement with the hypothesis that sibling competition has driven the evolution of visual nestling conspicuousness in a context of parent-offspring communication in altricial birds, such as had been previously shown for begging vocal displays.

## Supporting Information

File S1 Level of brood reduction, body mass, clutch size, duration of nestling period from Perrins (1987), nesting habits, nest material used and visual system of species used in the analyses. Number of chicks and nests from which we obtained reflectance values of gape structures is also presented.
(0.17 MB DOC)

File S2 Phylogenetic relationship among species included in the analyses.
(0.09 MB DOC)
