# Supplementary material for: Sibling Competition and Conspicuousness of Nestling Gapes in Altricial Birds: A Comparative Study
Source: PLoS One. 2010 May 6;5(5):e10509. doi: 10.1371/journal.pone.0010509 (PMC2865545; doi:10.1371/journal.pone.0010509)
Supplement: File S1 — Level of brood reduction, body mass, clutch size, duration of nestling period from Perrins (1987), nesting habits, nest material used and visual system of species used in the analyses. Number of chicks and nests from which we obtained reflectance values of gape structures is also presented. (0.17 MB DOC) [file pone.0010509.s001.doc]

Appendix 1. Level of brood reduction, body mass, clutch size, duration of nestling period from Perrins (1987), nesting habits, nest material used and visual system of species used in the analyses. Number of chicks and nests from we obtained reflectance values of gape structures is also presented.

| **Species** | **(%) Brood reduction** | **Literature (Brood reduction)** | **Clutch size** | **Nestling period (days)** | **Nesting habits** | **Body mass** | **N**  **Chicks (nests)** | **Nest material straw** | **Visual system** |
| --- | --- | --- | --- | --- | --- | --- | --- | --- | --- |
| *Athene noctua* | 13.2 | Cramp (1998)  Tomé et al. (2008) | 3 | 33 | Hole | 160.0 | 12 (3) | Ground | VS |
| *Columba libia* | 20.9 | Cramp (1998)  Johnston and Johnston (1990) | 2 | 36 | Hole | 302.0 | 4 (2) | Branches | VS |
| *Columba palumbus* | 28.5 | Cramp (1998) (two Pop.)  Slater (2001) | 1.5 | 34 | Non-hole | 496.0 | 2 (1) | Branches | VS |
| *Coracias garrulus* | 4.1 | Aviles and Parejo (2004) | 4 | 27 | Hole | 146.0 | 22 (5) | Ground | VS |
| *Corvus corone* | 35.5 | Cramp (1998)  Canestrari et al. (2008) | 4.5 | 31 | Non-hole | 561.0 | 12 (4) | Wool | VS |
| *Corvus modedula* | 63.9 | Soler (1990)  Soler and Soler (1996) | 5 | 33 | Hole | 256.0 | 15 (3) | Wool | VS |
| *Falco tinnunculus* | 22.5 | Cramp (1998) (three Pop.) | 4.5 | 30 | Hole | 156.0 | 13 (3) | Ground | VS |
| *Hirundo rustica* | 8.3 | Cramp (1998)  Møller (1994) | 4.5 | 21 | Hole | 19.0 | 13 (3) | Wool | UVS |
| *Lanius meridionalis* | 38.4 | Cramp (1998)  Antczak et al. (2004)  Campos et al. (2007) | 5 | 20 | Non-hole | 63.4 | 9 (2) | Straw | VS |
| *Lanius senator* | 36.3 | Cramp (1998)  Nikolov (2005) | 5.5 | 20 | Non-hole | 34.6 | 3 (1) | Straw | VS |
| *Oenanthe leucura* | 8.00 | Soler et al. (1995) | 4 | 14 | Hole | 44.0 | 26 (6) | Straw | UVS |
| *Otus scops* | 13.4 | Blanco et al. (2002) | 4.5 | 25 | Hole | 92.0 | 17 (4) | Ground | VS |
| *Parus major* | 17.8 | Amundsen and Slagsvold (1998)  Perrins (1965)  Pimentel and Nilsson (2007) | 8.5 | 20 | Hole | 18.9 | 15 (2) | Wool | UVS |
| *Passer domesticus* | 40.5 | Cramp (1998)  Lowther (1990) | 4.5 | 15 | Hole | 31.0 | 20 (4) | Straw | UVS |
| *Passer montanus* | 48.8 | Cramp (1998)  Cordero and Salaet (1990) | 4.5 | 13 | Hole | 20.8 | 20 (4) | Straw | UVS |
| *Petronia petronia* | 38.8 | Cramp (1998) (three Pop.) | 5.5 | 21 | Hole | 29.0 | 21 (6) | Straw | UVS |
| *Pica pica* | 18.3 | Cramp (1998) (three Pop.)  Ponz and Gil-Delgado (2004)  Birkhead (1991) | 6 | 25 | Non-hole | 234.6 | 19 (6) | Straw | VS |
| *Pyrrhocorax pyrrhocorax* | 43.8 | Kerviriou et al. (2006)  Cramp (1998) | 4 | 38 | Hole | 350.0 | 7 (2) | Wool | VS |
| *Sturnus unicolor* | 39.5 | Veiga (2002)  Soler et al. (Unpublished) | 4.5 | 22 | Hole | 90.0 | 242 (79) | Straw | UVS |
| *Turdus merula* | 12.1 | Paradis et al. (2000)  Preault et al. (2005) | 4 | 14 | Non-hole | 105.9 | 10 (4) | Straw | UVS |
| *Upupa epops* | 49.0 | Martín-Vivaldi et al. (1999) | 7.5 | 28 | Hole | 77.0 | 16 (2) | ground | VS |
