# Supplementary figures and images for: Sibling Competition and Conspicuousness of Nestling Gapes in Altricial Birds: A Comparative Study
Source: PLoS One. 2010 May 6;5(5):e10509. doi: 10.1371/journal.pone.0010509 (PMC2865545; doi:10.1371/journal.pone.0010509)

Appendix 2. Phylogenetic relationship among species included in the analyses


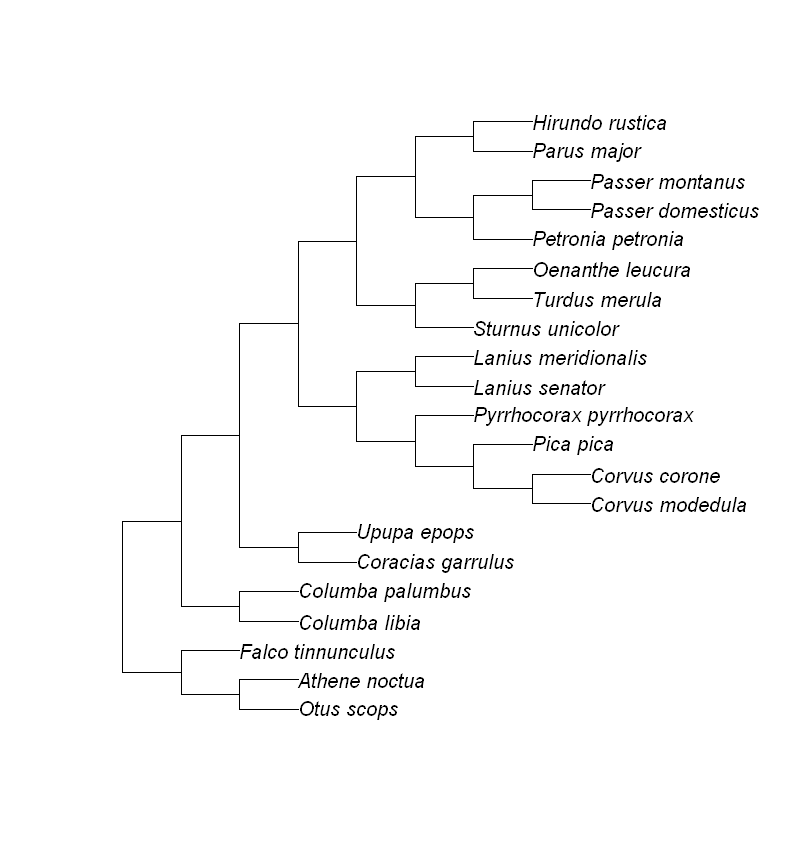

Supplement: File S2 — Phylogenetic relationship among species included in the analyses. (0.09 MB DOC) [file pone.0010509.s002.doc]
